# Supplementary material for: Clinical data and reporting quality in NMDAR-antibody encephalitis and pregnancy: a systematic review
Source: BMJ Neurol Open. 2025 Mar 3;7(1):e001005. doi: 10.1136/bmjno-2024-001005 (PMC11877225; doi:10.1136/bmjno-2024-001005)
Supplement: online supplemental file 1 [file bmjno-7-1-s001.pdf]

Supplementary material

Supplementary_Table_1.xlsx

Supplementary Table 1: Extracted data from published record according to idealised reporting checklist. Data frame of extracted data from included records is given in the first tab. Each pregnancy is reported on a separate row. Excluded full text articles are listed in the second tab.

**Reason For Exclusion**	**Search date**		
	**21/3/23**	**18/7/23**	**19/10/23**
Basic Science/Animal Study	23		1
Book Chapter	8		
Comment piece/Editorial	33	1	1
Non-pregnant/male cases	66	1	3
Not in English	13		
Other	6		
Paediatric Case/Study	472	26	13
Review Article	160	8	3
Study – Comparison Paediatric and Adult Cases	9		
Study – Cerebrospinal fluid contents	27		1
Study – Epidemiological	6		
Study – Genetics	2		
Study – Imaging	16		3
Study – Laboratory Diagnosis	13		
Study – Presenting/Clinical Features	42		1
Study – Scoring Systems	6		1
Study – Serum	11	1	1
Study – Treatment	11		2
Wrong disease	4	2	1

Supplementary Table 2: Reason for exclusion of records in initial and subsequent searches – number of records stated under date of corresponding search.

Study	Case Type	Outcome	Details
Chan et al.	During	Miscarriage	Presented in first trimester, miscarried within two days of hospitalisation.
Pennington et al.	During	Miscarriage	Miscarriage in first trimester whilst in early stages of disease.
Kim et al.	During	Miscarriage	Miscarried whilst in ICU, cause unclear.
Zhang et al.	During	Miscarriage	Miscarriage due to uterine haemorrhage at 16 weeks. Second presentation of NMDAR-Ab-E in pregnancy.
Reisz et al.	During	Stillbirth	Stillbirth at 27 weeks, in context of sepsis with pyothorax secondary to central venous catheter.
Keskin et al.	During	Stillbirth	Vaginal bleeding and hypotension at 32 weeks. US showed fetal demise and mother subsequently died from sepsis.
Kumar et al.	During	Termination	Termination whilst intubated, patient unable to consent.
Liu et al.	During	Termination	NMDAR-Ab-E during third pregnancy. Termination at 15 weeks. This was during the acute phase of the illness and family provided consent.
		Termination	NMDAR-Ab-E during fourth pregnancy. Termination at 15 weeks. Again, this was during the acute phase of the illness.
Kittichanteera et al.	During	Termination	Unexpected first trimester pregnancy discovered. Family gave consent for termination, patient acutely unwell and unable to consent.
Kokubun et al.	During	Termination	Patient in coma, family gave consent for termination.
Tian Nie et al.	During	Termination	Patient recovered sufficiently to consent to termination.
Wallengren et al.	Before	Termination	Accidental pregnancy after recovery, patient decision for termination.

Supplementary Table 3: Circumstances of miscarriage, stillbirth, and terminations identified within reported literature

Abbreviations: ICU=intensive care unit, NMDAR-Ab-E=NMDAR-antibody encephalitis, US=ultrasound

Study	Case Type	Details
Jagota et al.	During	Sepsis secondary to aspiration pneumonia. Treated with methylprednisolone and IVIG. Immunotherapy limited by infection.
Keskin et al.	During	Sepsis secondary to fetal demise. Treated with methylprednisolone and plasmapheresis. Immunotherapy limited by infection.
Kittichanteera et al.	During	Sepsis after initial recovery following treatment with IVIG and methylprednisolone.
Chen et al.	After	Multi-organ failure in context of severe illness not responsive to IVIG, methylprednisolone, rituximab, plasmapheresis and removal of ovarian teratoma.

Supplementary Table 4: Circumstances of maternal death identified within reported literature

Abbreviations: IVIG=intravenous immunoglobulin

Report	Illness onset relative to pregnancy	Neonatal Outcome	NMDAR-IgG transfer tested?	Childhood Outcome
Lamale-Smith et al.	During	Respiratory and neuromuscular depression likely secondary to maternal drugs, SVT requiring digoxin.	Yes. Fetal cord blood positive (1:20).	“Doing well and developmentally appropriate”
Jagota et al.	During	Intermittent episodes of spontaneous fine movements controlled by phenobarbital.	Yes. Neonatal serum positive two days after birth, (1:450).	"Delayed in global development and experienced generalized seizures"
Kumar et al.	During	Low AGPAR score, 3 at 1 minute, 6 at 5 minutes.	Yes. Not detected in neonatal serum, CSF or cord blood.	"Met all developmental milestones to date"
Scorrano et al.	During	Respiratory distress, hypoglycaemia. Brain ultrasound imaging showed periventricular hyper-echogenicity and sacral ultrasound imaging documented spina bifida. CXR showed right middle and lower lobe and left lower lobe consolidation.	Not Stated	Childhood outcome not stated.
Joubert et al.	During	Respiratory insufficiency recovering spontaneously at 24 hours, presumed secondary to maternal drugs.	Not Stated	"Normal behaviour and development"
Chourasia et al.	Before	hypotonia, poor respiratory efforts requiring intubation and ventilation, diffuse encephalopathy on EEG. MRI showed diffuse cerebral oedema and extensive ischaemic and haemorrhagic injury. Given IVIG for five days.	Yes. Neonatal serum positive (1:320).	Neonatal death
Hilderink et al.	Before	Respiratory insufficiency. Moro, rooting, grasp, suck and swallow reflexes decreased but muscle tone normal. Required oxygen and gastric tube feeding. IV anti-biotics for 3 days. Opened eyes at day 6 and began feeding, feeding problems resolved by day 10.	Yes. Neonatal serum positive three days after birth (~1:400).	"At 12 months development was normal"

Supplementary Table 5: Details and outcomes of compromised neonates identified within reported literature. NMDAR-IgG assay end-point dilution stated in the report is noted.

Abbreviations: APGAR=appearance, pulse, grimace, activity and respiration, CXR=chest x-ray, CSF=cerebrospinal fluid, EEG=electroencephalogram, IVIG=intravenous immunoglobulin, NMDAR-IgG=Immunoglobulin G autoantibody against N-Methyl D-Aspartate receptor, SVT=supraventricular tachycardia

Case	Disease onset relative to pregnancy	Age range at NMDAR-Ab-E onset (years)	Ovarian teratoma?	Age range at pregnancy onset	Conception	Trimester at NMDAR-Ab-E onset	Total admission duration range (months)	Delivery time days post-NMDAR-Ab-E onset (months)	Pregnancy outcome	Mode of delivery	Gestational age (Term or Preterm)
Case 1 Pregnancy 1	During	31-35	Yes	31-35	Spontaneous	2nd	<6	<1	Livebirth	EMCS	Preterm
Case 2 Pregnancy 1	Before	31-35	Yes	31-35	Spontaneous	N/A	<2	N/A	Livebirth	Spontaneous vaginal delivery	Term
Case 2 Pregnancy 2	Before	As above	Yes	36-40	Spontaneous	N/A	As above	N/A	Livebirth	EMCS	Term
Case 3 Pregnancy 1	Before	21-25	Yes	26-30	Spontaneous	N/A	<1	N/A	Miscarriage	N/A	N/A
Case 3 Pregnancy 2	Before	As above	As above	26-30	Spontaneous	N/A	As above	N/A	Livebirth	Spontaneous vaginal delivery	Preterm
Case 3 Pregnancy 3	Before	As above	As above	26-30	Spontaneous	N/A	As above	N/A	Livebirth	Spontaneous vaginal delivery	Term

Supplementary Table 6: Maternal demographics, NMDAR-Ab-E presentation, pregnancy timings and key outcomes. Abbreviations: EMCS = emergency C-section, GxPx=gravidity and parity, N/A=not applicable, NMDAR-Ab-E=NMDAR-antibody encephalitis

Case	Initial MRI brain abnormal?	Initial EEG abnormal?	Initial CSF protein raised?	Initial CSF cell count raised?	Serum NMDAR-IgG (initial)	CSF NMDAR-IgG (initial)	Ovarian teratoma identified?	ICU admission	Immuno-therapy and/or oophorectomy during illness?	If RTX, was it pre- or post-delivery?	Symptomatic treatments	Maternal outcome	Length of maternal follow-up since most recent illness episode (years)
Case 1 Pregnancy 1	Yes	Yes	No	Yes	Positive	Positive	Yes	Yes	Immunotherapy Oophorectomy	Post-delivery	Anti-seizure medication Anti-psychotic medication	Outpatient neuro-rehabilitation	1
Case 2 Pregnancy 1	No	Yes	No	Yes	Positive	Positive	Yes	Yes	Immunotherapy Oophorectomy	NA	Anti-seizure medication Anti-psychotic medication	Recovered, no deficit	7
Case 2 Pregnancy 2	NA	NA	NA	NA	NA	NA	NA	NA	As above	NA	NA	Recovered, no deficit	As above
Case 3 Pregnancy 1	Yes	N/A	N/A	N/A	Positive	Positive	Yes	No	Immunotherapy Oophorectomy	N/A	Anti-seizure medication Anti-psychotic medication	Recovered, no deficit	7
Case 3 Pregnancy 2	As above	As above	As above	As above	Seronegative during pregnancy	As above	As above	As above	As above	As above	As above	As above	As above
Case 3 Pregnancy 3	As above	As above	As above	As above	Seronegative during pregnancy	As above	As above	As above	As above	As above	As above	As above	As above

Supplementary Table 6: NMDAR-Ab-E investigations, treatment and maternal outcomes. Abbreviations: CBA=cell-based assay, CSF=cerebrospinal fluid, CT=computed tomography, EEG=electroencephalogram, MRI= magnetic resonance imaging, N/A=not applicable, NMDAR-IgG=Immunoglobulin G autoantibody against N-Methyl D-Aspartate receptor, PLEX=plasma exchange, RTX=rituximab, US=ultrasound

Case	Fetal growth restriction	Hypertensive disorder of pregnancy	Gestational diabetes	Ante-partum haemorrhage	Low-lying placenta	Ovarian cyst accident	Emergency surgery during pregnancy	Preterm or Term birth	Cord prolapse	Placental abruption	Meconium aspiration	Chorio-amnionitis	Post-partum haemorrhage (>500ml)	Blood transfusion	Delivery under GA
Case 1 Pregnancy 1	No	Yes	Yes	No	No	No	No	Preterm	No	No	No	No	No	No	Yes
Case 2 Pregnancy 1	No	No	Yes	No	No	No	No	Term	No	No	No	No	Yes	No	No
Case 2 Pregnancy 2	No	No	Yes	No	No	No	No	Term	Yes	No	No	No	Yes,	No	No
Case 3 Pregnancy 1	N/A	N/A	N/A	N/A	N/A	N/A	N/A	N/A	N/A	N/A	N/A	N/A	N/A	N/A	N/A
Case 3 Pregnancy 2	No	No	No	No	No	No	No	Preterm	No	No	No	No	No	No	No
Case 3 Pregnancy 3	No	No	No	No	No	No	No	Term	No	No	No	No	No	No	No

Supplementary Table 6: Antenatal and delivery outcomes. Abbreviations: GA=general anaesthetic, N/A=not applicable, NMDAR-Ab-E=NMDAR-antibody encephalitis, PPROM=preterm premature rupture of membranes.

Case	Testing of placenta or neonate for NMDAR-IgG	Neonatal condition	Birth weight (nearest kg)	Normal APGARs by 10 minutes?	Normal cord gas?	HIE?	SCBU admission	Cooling	Infection	Seizures	Breast feeding before discharge	Childhood condition – general statement	Milestones – on target or delayed	Medical diagnoses	Neurologic diagnoses	ASD or ADHD	Any behaviour condition	Length of child follow-up (years)
Case 1 Pregnancy 1	No	Apnoeic episodes<br>Bradycardia<br>Jaundice<br>Ascites	2	Unavailable	Unavailable	No	Yes	No	No	No	Yes	Good	On target	No	No	No	No	1
Case 2 Pregnancy 1	Both detectable on live CBA	TTN	4	Yes	Unavailable	No	No	No	No	No	No	Good	On target	No	No	No	No	5
Case 2 Pregnancy 2	No	Good	4	Yes	Normal	No	No	No	No	No	Yes	Good	On target	Eczema	No	No	No	1.5
Case 3 Pregnancy 1	No	N/A	N/A	N/A	N/A	N/A	N/A	N/A	N/A	N/A	N/A	N/A	N/A	N/A	N/A	N/A	N/A	N/A
Case 3 Pregnancy 2	No	Low APGARs<br>Jaundice<br>Sepsis	3	Yes	Arterial - Abnormal	No	Yes	No	Yes	No	Yes	Good	On target	No	None	Under review	No	4
Case 3 Pregnancy 3	No	Good	3	Yes	Unavailable	No	No	No	No	No	Yes	Good	On target	No	No	No	No	2

Supplementary Table 6: neonatal and childhood outcomes. Abbreviations: APGAR=appearance, pulse, grimace, activity and respiration, ASD=autistic spectrum disorder, ADHD=attention deficit hyperactivity disorder, BE=base excess, CBA=cell-based assay, CRP=C-reactive protein, HIE=hypoxic ischaemic encephalopathy, IV=intra-venous, N/A=not applicable, NMDAR-IgG=Immunoglobulin G autoantibody against N-Methyl D-Aspartate receptor, PN=parenteral nutrition, PPROM=preterm premature rupture of membranes, SCBU=special care baby unit, TTN=transient tachypnoea of the newborn

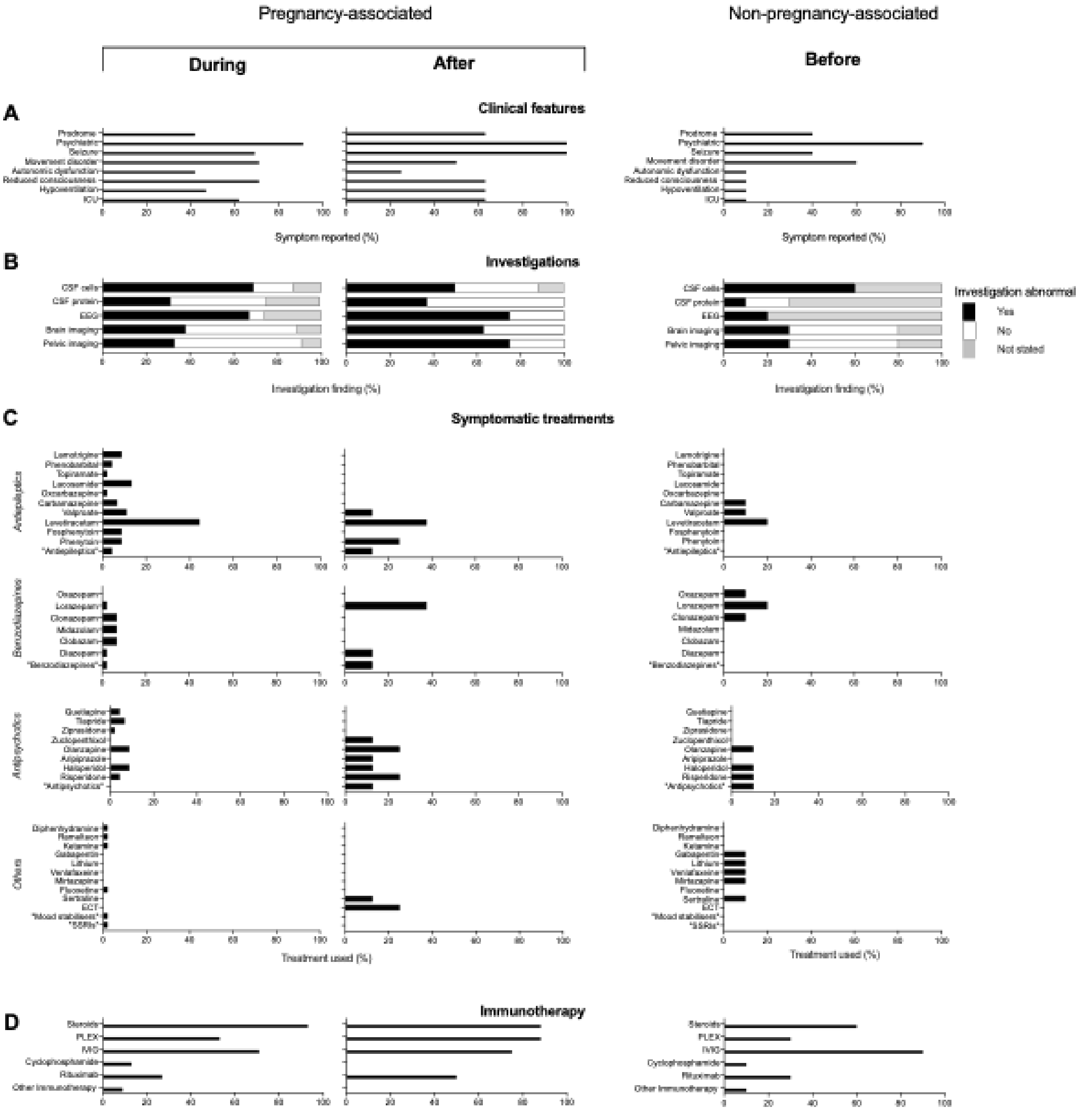

Supplementary Figure 1: NMDAR-Ab-E clinical features and treatments according to pregnancy association

A – Rates of clinical features in each group indicated by percentage. B – Rates of investigations and outcomes (abnormal=black, not abnormal=weight; uncertain if investigation done=grey). Brain imaging includes CT or MRI and pelvic imaging includes CT, US, or MRI. C – Rate of symptomatic treatments into anti-epileptics, benzodiazepines, anti-psychotics and other. D – Rates of immunotherapy. Other immunotherapy: during – azathioprine (2), and tacrolimus (1) and bortezomib (1); before – azathioprine (1).

Abbreviations: CT=computed tomography, CSF=cerebrospinal fluid, ECT=electroconvulsive therapy, EEG=electroencephalogram, ICU=intensive care unit, IVIG=intravenous immunoglobulin, MRI=magnetic resonance imaging, PLEX=plasma exchange, SSRI=selective serotonin reuptake inhibitor, US=ultrasound.

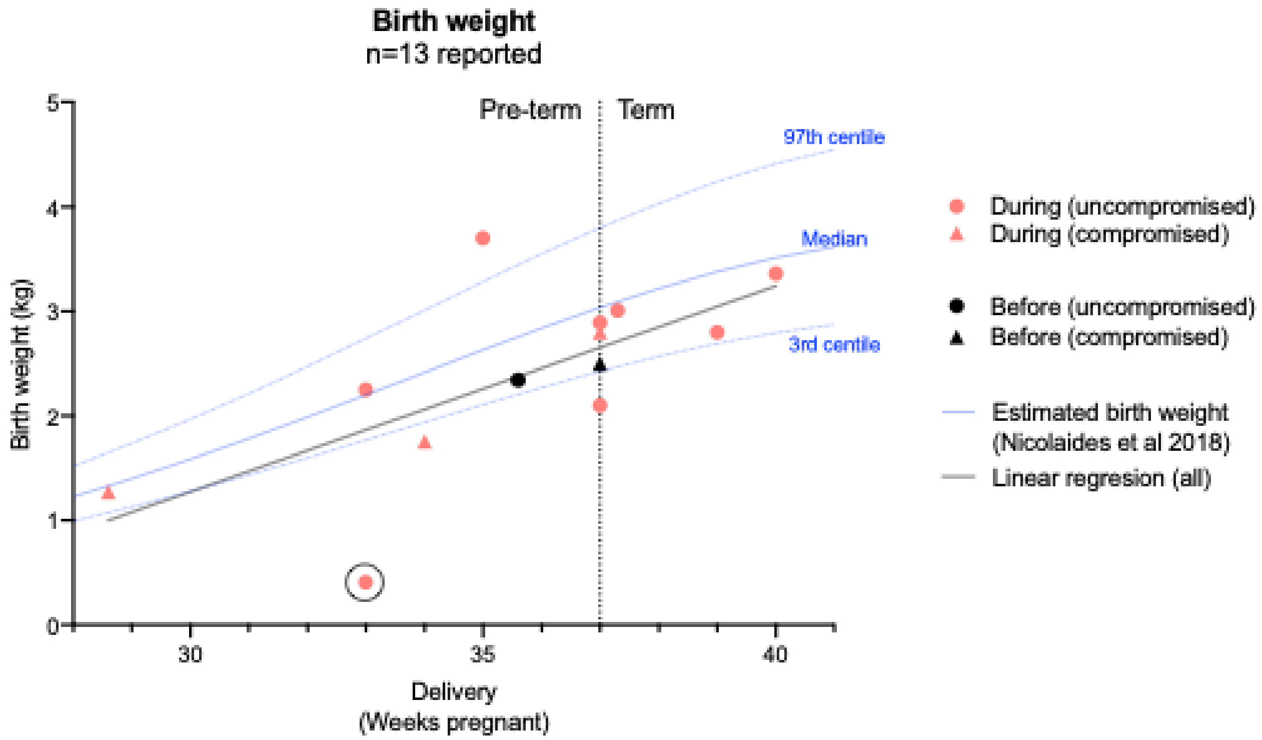

Supplementary Figure 2: Neonatal birthweights including outlying value

The plot from Fig. 3C is reproduced to include and analyse an outlying reported value of 408g at 33 weeks (circled) [22]. This weight is extremely low for gestational week 33 and premature babies born this small would be unlikely to survive [23]. Attempts to contact authors to clarify birthweight were unsuccessful. The line of best fit consequently moves towards the lower limit of normative values.

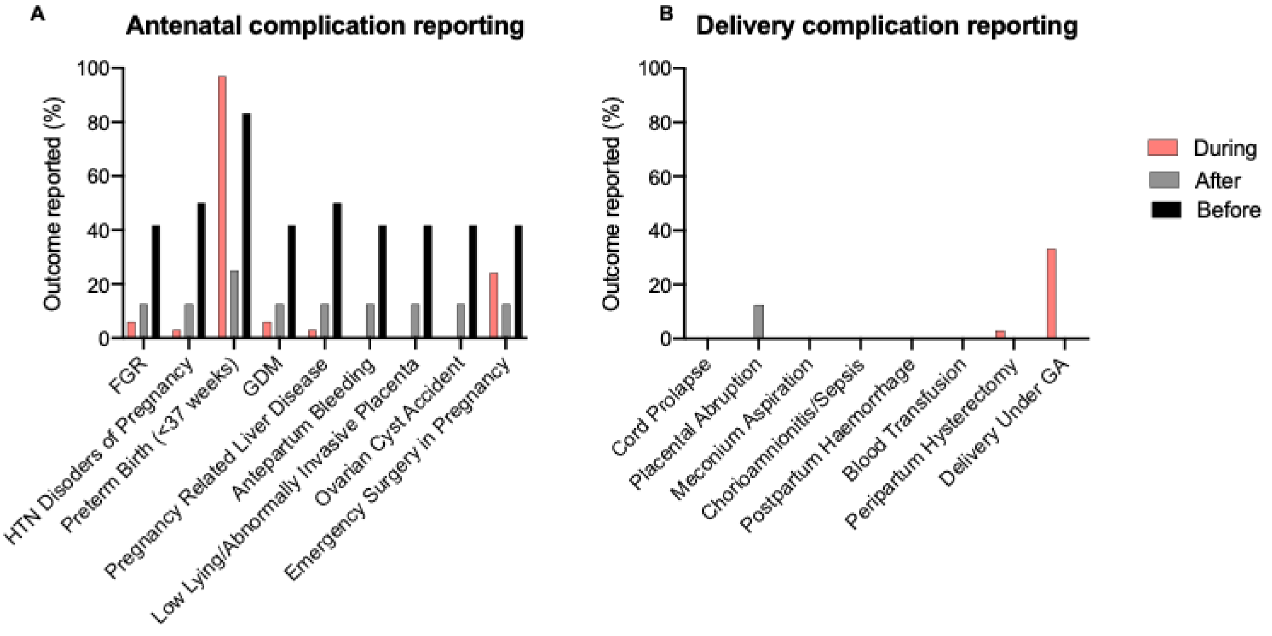

Supplementary Figure 3: Antenatal and delivery outcome reporting

A – Percentage of each case type reporting on each of the listed antenatal complications, either positively or negatively. B – Percentage of each case type reporting on each of the listed delivery complications, either positively or negatively.

Abbreviations: FGR=fetal growth restriction, GA=general anaesthetic, GDM=gestational diabetes mellitus, HTN=hypertensive

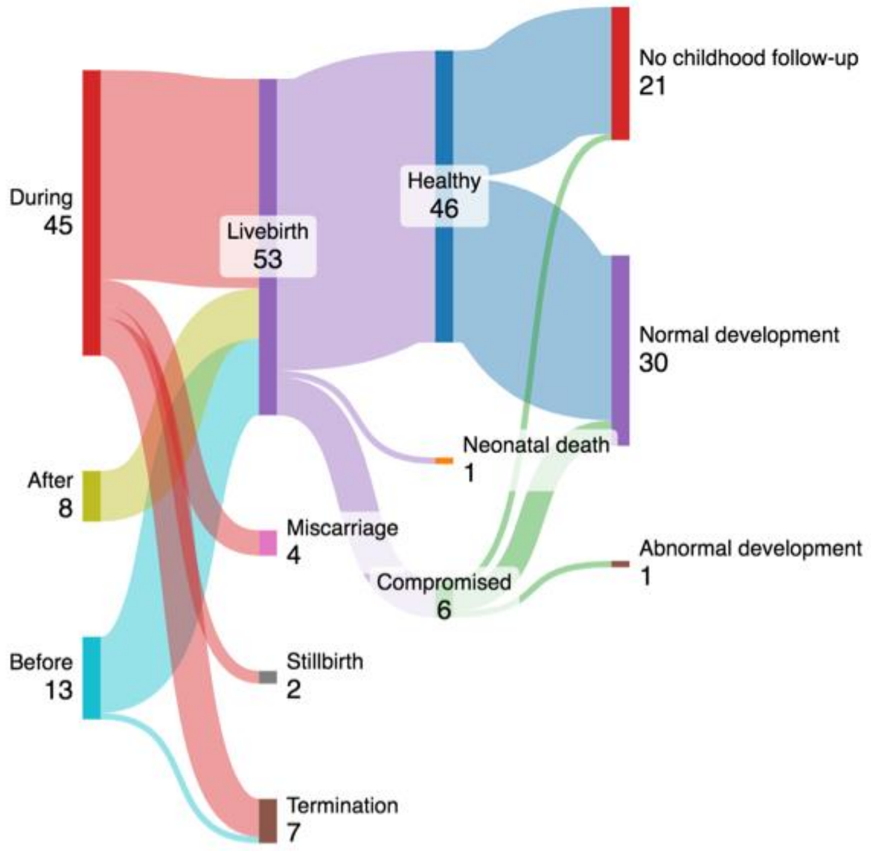

Supplementary Figure 4: Outcomes summary

Sankey diagram showing relationship between pregnancy, neonatal and childhood outcomes in mothers who had NMDAR-antibody encephalitis before, during, or after a pregnancy. Numbers of pregnancies are given then after birth numbers relate to offspring.
